# Early and mid-term outcomes after aortic valve intervention in patients with previous stentless or stented bioprostheses

**DOI:** 10.1186/s13019-023-02118-3

**Published:** 2023-01-18

**Authors:** Naoto Fukunaga, Ali Al-Sarraf, Khalil Jawad, Myriam Lafreniere-Roula, Vivek Rao

**Affiliations:** Division of Cardiovascular Surgery, 4PMB-457, Peter Munk Cardiac Centre, Toronto General Hospital, University of Toronto, University Health Network, 200 Elizabeth Street, Toronto, ON M5G 2C4 Canada

**Keywords:** Redo aortic intervention, Stented valve, Stentless valve

## Abstract

**Background:**

Limited data are available concerning comparative outcomes of redo aortic valve interventions, including surgery after aortic valve replacement (AVR) with either stented or stentless bioprostheses. We investigated the comparative outcomes of redo aortic valve interventions, including surgery after AVR with either stented or stentless bioprostheses.

**Methods:**

The cohort consisted of 112 patients who underwent aortic valve intervention for infective endocarditis or structural valve deterioration between 2001 and 2020. One hundred patients received a stented valve (stented group) and 12 patients received a stentless valve (stentless group) during the initial surgery. Early and late outcomes were evaluated.

**Results:**

The mean [IQR] ages during the current interventions were 66 [54, 77] years in the stented group and 74 [67, 79] years in the stentless group (*P* = 0.13). In the stented group, aortic valve interventions included redo AVRs with stented valves (n = 54), mechanical valves (n = 26), stentless valves (n = 16), and transcatheter aortic valve implantations (n = 4). In the stentless group, redo AVRs were performed with stented valves (n = 4), mechanical valves (n = 2), stentless valves (n = 1), and transcatheter valve implantations (n = 5). Hospital mortality was observed in 2 (2%) patients in the stented group and 1 (8%) patients in the stentless group (*P* = 0.29). The 5-year survival was 80.8% [66.8, 88.5] in the stented group and 91.7% [53.9, 98.8] in stentless group. Statistically significant differences in thromboembolisms were observed between the groups.

**Conclusions:**

No significant differences in early and mid-term outcomes (except thromboembolism) after aortic valve interventions were detected between patients with stented and stentless AVRs.

## Introduction

The number of aortic valve replacements (AVRs) is increasing. The use of a bioprosthesis increased from 37.7% in 1998–2001 to 63.6% in 2007–2011. This trend was particularly dramatic in patients aged 55 to 64 years [1]. With the increase utilization of bioprostheses, increasing cases of redo interventions are expected. Even without the utilization of transcatheter technique, the number of redo AVRs for structural valve deterioration (SVD) has been increasing [2]. In addition, patients’ age has increased with low mortality rates [3].

Operative mortality following redo AVRs for SVD is 3.5–6.8% [2, 4], which seems reasonable. However, the mortality rate following redo AVRs for prosthetic valve endocarditis (PVE) is over 20%, with poor late survival [4]. When surgeries are reperformed following AVRs with stentless bioprostheses, the mortality rate approaches 21% [5–7]. An analysis of 57 patients who underwent redo surgery for stentless SVD (84%) and PVE (16%) by Borger, et al. showed that redo surgery after stentless AVR caused trauma to the coronary ostia, aortic wall/annulus, anterior mitral leaflet, and membranous septum. Root replacement was frequently required for the patient cohort [5]. After previous cardiac surgery, indirect coronary reimplantation was a risk factor for increased 30-day mortality for the Bentall procedure [8]. In the current era, transcatheter valve-in-valve procedures are available instead of the conventional open surgery. Limited data are available concerning comparative outcomes of redo aortic valve interventions, including surgery after AVR with either stented or stentless bioprostheses. Therefore, we investigated early and mid-term outcomes of redo aortic valve interventions between patients with previous stentless or stented bioprostheses.

## Methods

The institutional review board approved this study. A consent form was waived in the nature of a retrospective study.

The patient cohort consisted of 112 patients who underwent aortic valve intervention for PVE or SVD between 2001 and 2020; 100 patients received stented bioprostheses (stented group) and 12 patients received stentless bioprostheses (stentless group) during their initial surgery. PVE only occurred in the stented group (n = 27). Early and mid-term outcomes including survival, reoperation, thromboembolism, endocarditis, and pacemaker insertion were evaluated. Indications of redo intervention were as follows. If patients were tolerated with redo open surgery with reasonable risk analysis, the patients went to redo open surgery. If patients were old, looked frail, or had the high mortality and morbidity rates, they went to transcatheter valve-in-valve procedures.

This study was conducted according to the guidelines for reporting mortality and morbidity after cardiac valve interventions [9].

The median duration of follow-up was 3.5 [0.4, 7.1] years. The follow-up rate was 94.6%.

### Statistical analysis

Baseline clinical and surgical characteristics were summarized using descriptive statistics. Continuous variables were summarized in terms of median and interquartile range (IQR) with the IQR being the range between the first and third quartile. Dichotomous and polytomous variables were summarized in terms of frequencies and proportions. Databases used for data collection changed over the study period and variables were matched across collection eras as best as possible. However, biases were not excluded in the nature of the retrospective analysis without propensity matched score. Mid-term outcomes of interest included mortality, valve reoperation, thromboembolism, endocarditis, and pacemaker insertion. Survival estimates were generated using the Kaplan–Meier method. The cumulative proportion of valve reoperation was estimated using a competing risk model with death as the competing risk. Cumulative proportion estimates for other long-term outcomes were estimated using competing risk models with death or valve reoperation as the competing risk.

## Results

Mean [IQR] ages during the current intervention were 66 [54, 77] years old in the stented group and 74 [67, 79] years old in the stentless (*P* = 0.13). Most patients in both groups presented with NYHA class III/IV symptoms (*P* = 0.19). Over 80% of patients had grade I/II left ventricular function, including > 40% of patients preoperatively (*P* = 0.84). No significant differences in preoperative comorbidities were observed. Patients’ characteristics are summarized in Tables 1 and 2.

**Table 1:** Patients’ characteristics

Variable	N—Stented	Stat—Stented	N—Stentless	Stat—Stentless	*P* value
Age at time of surgery (yrs)	100	66 (54–77)	12	74 (67–79)	0.135
Sex of patient	100		12		0.181
Female		28 (28%)		1 (8%)	
Male		72 (72%)		11 (92%)	
Diabetes Mellitus (insulin or non-insulin dependent)	100		12		1.00
Yes		18 (18%)		2 (17%)	
Medically treated hypertension	100		12		0.35
Yes		57 (57%)		9 (75%)	
Diet or medically treated hyperlipidemia	100		12		0.76
Yes		58 (58%)		6 (50%)	
Chronic obstructive pulmonary disease—severe	100		11		0.41
Yes		4 (4%)		1 (9%)	–
Previous stroke or T.I.A	100		12		0.69
Yes		19 (19%)		1 (8%)	
Evidence of any atherosclerotic lesion outside of the heart and proximal aorta	100		12		0.69
Yes		8 (8%)		2 (17%)	
Pre-op dialysis	100		12		0.29
Yes		1 (1%)		1 (8%)	
Family history of heart disease including stroke, PVD, MI, angina, or heart surgery	100		11		0.20
Yes		47 (47%)		4 (36%)	0.58
Smoking history	100		12		1.00
Never smoked		45 (45%)		6 (50%)	
Still smoking		7 (7%)		0 (0%)	
Stopped smoking		48 (48%)		6 (50%)	1.00
Pre-op atrial fibrillation/flutter	100		12		1.00
Yes		15 (15%)		2 (17%)	
Pre-op complete heart block	100		12		1.00
Yes		9 (9%)		0 (0%)	
Congestive Heart Failure	100		12		0.59
Yes		66 (66%)		8 (67%)	
Shock	100		12		1.00
Yes		1 (1%)		1 (8%)	
Syncopal episodes	100		12		0.20
Yes		2 (2%)		0 (0%)	
Aortic valve disease	100		11		1.00
Mixed		26 (26%)		2 (18%)	
None		7 (7%)		1 (9%)	
Regurgitation (greater than mild: > 2 +)		44 (44%)		4 (36%)	0.66
Stenosis		23 (23%)		4 (36%)	
Mitral valve disease	100		12		
Missing		1 (1%)		0 (0%)	
Mixed		2 (2%)		0 (0%)	
None		85 (85%)		12 (100%)	0.54
Regurgitation (greater than mild: > 2+)		12 (12%)		0 (0%)	
Tricuspid valve disease	100		12		
None		94 (94%)		12 (100%)	
Regurgitation (greater than mild: > 2+)		5 (5%)		0 (0%)	
Stenosis		1 (1%)		0 (0%)	1.00

**Table 2:** Clinical presentations

Variable	N—Stented	Stat—Stented	N—Stentless	Stat—Stentless	*P* value
Most severe angina pectoris within one month prior to surgery	100		12		0.30
Acute coronary insufficiency: prolonged episodes of unprovoked pain (> 15 min) despite medical therapy		1 (1%)		1 (8%)	
Crescendo: increasing frequency or severity of symptoms		2 (2%)		0 (0%)	
None		83 (83%)		9 (75%)	
Stable: predictable exertional angina		14 (14%)		2 (17%)	
Infective endocarditis	99		11		0.51
Active		5 (5%)		0 (0%)	
Active abscess		9 (9%)		0 (0%)	
Remote		13 (13%)		0 (0%)	
Most recent myocardial infarction within 30 days of OR date	99		8		0.090
Non-Q wave infarction		3 (3%)		0 (0%)	
Transmural infarction		0 (0%)		1 (12%)	
NYHA classification (cardiac disability)	100		12		0.190
No restrictions		11 (11%)		4 (33%)	
Symptoms provoked by exertion beyond daily activity		10 (10%)		0 (0%)	
Symptoms provoked by normal daily activity		41 (41%)		4 (33%)	
Unprovoked symptoms		38 (38%)		4 (33%)	
LV grade based on LV ejection fraction	100		12		0.84
< 20%		1 (1%)		0 (0%)	
> = 60%		46 (46%)		7 (58%)	
20–39		11 (11%)		1 (8%)	
40–59		42 (42%)		4 (33%)	

In the stented group, aortic valve interventions included redo aortic valve surgery with stented valves (n = 54), mechanical valves (n = 26), stentless valves (n = 16), and transcatheter aortic valve implantations (n = 4). Of the surgical cases, 41 patients (41%) required root replacement. In the stentless group, redo aortic valve surgery included stented valves (n = 4), mechanical valves (n = 2), a stentless valve (n = 1), and transcatheter valve implantations (n = 5). Half of the surgical cases (n = 6) was root replacements. Details in their explanted and newly implanted prosthetic valves are described in Table 3. Concomitant aortocoronary bypass grafting was performed in 31 patients (31%) in the stented group and 2 (17%) patients in the stentless group (*P* = 0.50). Data on concomitant procedures during initial and redo surgery are available on Table 4.

**Table 3:** Details in prosthetic valves

Variable	N—Stented	Stat—Stented	N—Stentless	Stat—Stentless	*P* value
Type of explanted prosthesis	100		12		< 0.001
Carpentier-Edwards Pericardial		7 (7%)		0 (0%)	
Carpentier-Edwards Pericardial		24 (24%)		0 (0%)	
Epic		1 (1%)		0 (0%)	
Freestyle stentless		0 (0%)		4 (33%)	
Hancock II Porcine		54 (54%)		0 (0%)	
Magna Pericardial		2 (2%)		0 (0%)	
Mitroflow		3 (3%)		0 (0%)	
Mosaic		8 (8%)		0 (0%)	
Toronto Stentless Porcine		0 (0%)		8 (67%)	
Trifecta		1 (1%)		0 (0%)	
Type of prosthesis implanted in current procedure	100		12		< 0.001
Carpentier-Edwards Pericardial		7 (7%)		0 (0%)	
Carpentier-Edwards Perimount Magna Ease		0 (0%)		1 (8%)	
Epic		1 (1%)		0 (0%)	
Freestyle Stentless		11 (11%)		1 (8%)	
Hancock II Porcine		37 (37%)		1 (8%)	
Homograft		5 (5%)		0 (0%)	
Magna Pericardial		6 (6%)		1 (8%)	
Medtronic Hancock II Aortic Cinch		0 (0%)		1 (8%)	
Mosaic		3 (3%)		0 (0%)	
St-Jude		26 (26%)		0 (0%)	
St. Jude HP Aortic Valved Graft		0 (0%)		1 (8%)	
St.Jude Aortic Valved Graft		0 (0%)		1 (8%)	
Trans-catheter-Apical		4 (4%)		1 (8%)	
Trans-catheter-Femoral		0 (0%)		4 (33%)

**Table 4:** Previous and current concomitant procedures

Variable	N—Stented	Stat—Stented	N—Stentless	Stat—Stentless	*P* value
*Previous concomitant procedures*					
Previous ACB	100		12		1.00
Yes		17 (17%)		2 (17%)	
Previous aortic valve surgery	100		12		–
Replacement		100 (100%)		12 (100%)	
Previous mitral valve surgery	100		12		0.78
Repair		9 (9%)		0 (0%)	
Replacement		5 (5%)		0 (0%)	
Previous tricuspid valve surgery	100		12		–
No		100 (100%)		12 (100%)	
Any other previous cardiac surgery	100		12		0.26
Yes		19 (19%)		4 (33%)	
*Current concomitant procedures*					
CABG procedure (in conjunction)	100		12		0.50
Yes		31 (31%)		2 (17%)	
Ascending aorta replaced	100		12		0.81
Yes, composite		41 (41%)		6 (50%)	
Yes, conduit separate from valve		8 (8%)		0 (0%)	
Aortic valve surgery	100		12		–
Replacement		100 (100%)		12 (100%)	
Mitral valve surgery	100		12		0.36
Repair		7 (7%)		0 (0%)	
Replacement		12 (12%)		0 (0%)	
Tricuspid valve surgery	100		12		1.00
Repair		7 (7%)		0 (0%)	
Replacement		1 (1%)		0 (0%)	
Repair of congenital defect	100		12		1.00
ASD		1 (1%)		0 (0%)	
VSD		2 (2%)		0 (0%)	
Hours on ventilator (from arrival in ICU till extubation time)	100	9.16 (5.08- 21.33)	12	4.1000 (0.0000- 16.8275)	0.032
Total number of hours in ICU (from arrival in ICU till departure to floor)	100	53.04 (23.52 -100.12)	12	69.95 (24.78- 116.71)	0.66

### Early outcomes

Hospital mortality was observed in three patients (2.7%). Two deaths (2%) occurred in the stented group and 1 (8%) death occurred in the stentless group (*P* = 0.29). In the stented group, one patient who underwent redone AVR and CABG for SVD died of respiratory failure. The second patient in the stented group had redo AVR combined with mitral valve replacement, tricuspid valve annuloplasty, replacement of the ascending aorta, and CABG and died of bleeding in the operating theater. In the stentless group, the death occurred in a patient who underwent a Bentall procedure for SVD and disease of bowel ischemia. No significant differences in postoperative complications, such as re-exploration for bleeding, perioperative myocardial infarction, new onset of atrial fibrillation, pulmonary complications, or sepsis, were detected between groups (Table 5).

**Table 5:** Perioperative outcomes

Variable	N—Stented	Stat—Stented	N—Stentless	Stat—Stentless	*P* value
Reop for bleeding/tamponade	100		12		0.29
Yes		8 (8%)		2 (17%)	
Postoperative electrocardiogram—ischemic changes while in hospital	99		12		1.00
New ST and T wave changes		3 (3%)		0 (0%)	
No changes		96 (97%)		12 (100%)	
Presence of atrial fibrillation post-operatively	100		12		0.119
Yes		43 (43%)		2 (17%)	
Pulmonary complications	100		12		1.00
Yes		18 (18%)		2 (17%)	
Seizures post-op	100		8		0.38
Yes		5 (5%)		1 (12%)	
Leg infection	100		8		–
No		100(100%)		8 (100%)	
Sepsis—based on positive blood culture	100		12		1.00
Yes		3 (3%)		0 (0%)	
Perioperative mortality	100		12		0.29
Alive		98 (98%)		11 (92%)	
Died		2 (2%)		1 (8%)	

### Mid-term outcomes

**Fig. 1:**
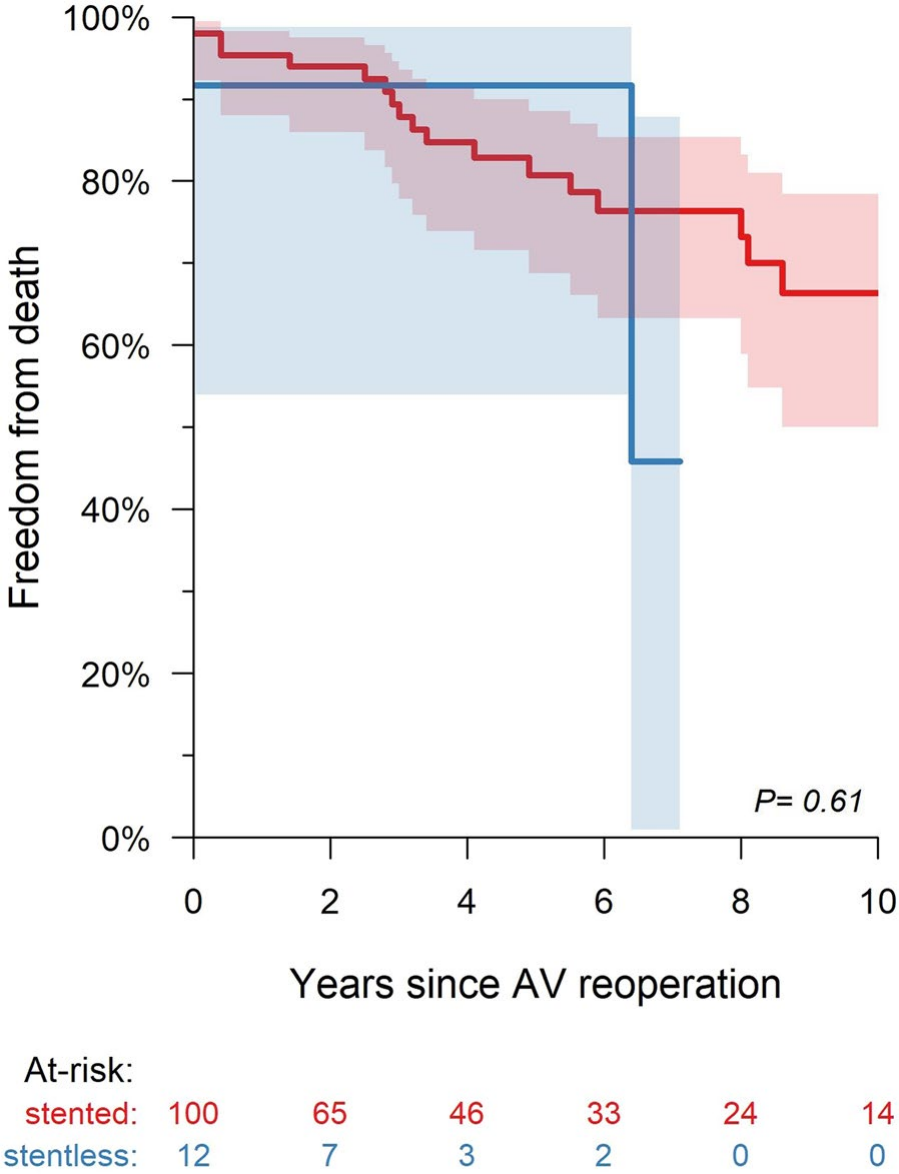
Survival rates after redo aortic valve intervention between stented and stentless groups. There was no statistically significant difference among groups

Overall survival rates were 95.0% [88.3, 97.9] at 1 year, 81.5% [70.2, 88.8] at 5 years, and 65.1% [49.1, 77.2] at 10 years. The 5-year survival rate in the stented group was 80.8% [66.8, 88.5] and 5-year survival rate in the stentless group was 91.7% [53.9, 98.8] (Log-rank *P* = 0.61) (Fig. 1). Freedom from the cumulative proportion of reoperation, thromboembolism, and pacemaker implantation at 10 years was 0%, 6.8% [2.1, 21.9], and 2.7% [0.7, 11.2], respectively, for the entire cohort. The rate of thromboembolisms was significantly higher in the stentless group compared with the rate in the stented group (0% in the stented group versus 9.2% [1.4, 59.3] in the stentless at 5 years, Log-rank *P* < 0.001). Of the three patients with thromboembolisms in the stentless group, one patient received AVR with a mechanical valve and the other two patients underwent Bentall procedures with tissue valves.

## Discussion

Except for thromboembolisms, we found no significant differences in early and mid-term outcomes after aortic valve interventions for SVD or PVE in patients with stented or stentless valves. Redo interventions for the stentless group were challenging because half the patients required Bentall procedures. Despite the lack of statistical differences in mortality between the groups, mortality in the stentless group was 8%. Furthermore, the stented group was arguably a higher risk group prior to reintervention.

Two types of bioprostheses are used in the aortic position. Both stented and stentless bioprostheses are available for various valvular diseases with hemodynamically satisfactory outcomes. Implantation techniques for stentless bioprostheses are more complicated compared with implantation of stented bioprostheses. Therefore, the use of stentless bioprostheses has been limited [10]. Instead, stented bioprostheses have been widely applied. The use of bioprostheses increased from 37.7% in 1998 to 2001 to 63.6% in 2007 to 2011 [1]. This trend is likely attributable to both patients’ preference to avoid anticoagulation and the emergence of the transcatheter valve-in-valve technique for SVD. The guidelines state that prosthetic selection should be a shared decision-making process that takes into account the patients’ values and preferences, with an understanding of the future need for interventions [11], which strongly affects patients’ preference.

Historically, redo valvular surgery had a high mortality rate of 7–10% [12–16]. Improvements of myocardial protection and surgical techniques, use of hypothermic circulatory arrest, peripheral cardiopulmonary bypass, and more experience with redo surgery contributed to improved outcomes in redo valvular surgery over the past few decades [3, 14].

The contemporary mortality rate after isolated redo AVR based on The Society of Thoracic Surgeons Adult Cardiac Surgery Database was 4.6% compared with 2.2% for primary AVR [17]. Redo AVR requires concomitant surgical procedures, such as coronary bypass grafting and mitral and/or tricuspid valve surgery, in 17% of the patients [18]. In our study, 41% of the patients required a Bentall procedure following AVR. In the current era, transcatheter valve-in-valve implantation is available, even for failed bioprostheses. Centers that are experienced in transcatheter aortic valve implantations have moved to valve-in-valve implantations for failed bioprostheses, given the risks and comorbidities of patients. However, a significant proportion of patients presented with contraindication for transcatheter intervention due to infection, mechanical aortic valve, and/or need for concomitant procedures [18]. These cases still require conventional cardiac surgery, which might be complex in some cases.

The mortality rate of redo AVRs following AVRs with stentless bioprostheses are as high as 21% [5–7]. The primary method of reoperation following stentless AVR requires complete explantation of the stentless bioprosthesis [6, 10], which may cause trauma to coronary ostia, aortic wall/annulus, anterior mitral leaflet, membranous septum, and require concomitant procedures. Over 60% of patients underwent redo aortic root replacements for failed stentless valves [5, 10]. Our experience was similar. An alternative to redo aortic root replacement is valve-in-valve implantation, whereby a stented valve is placed within the debrided stentless valve. The mortality rate after this technique is 3%, whereas the mortality rate for redo aortic root replacement is 11% [6].

Recent studies demonstrated that procedural mortality, 30-day mortality, major bleeding, and the rate of stroke were lower for valve-in-valve percutaneous implantations compared with redo AVRs [19–21]. These outcomes were expected, given the invasiveness in redo surgery. However, no significant difference in the rate of cardiovascular mortality, myocardial infarction, and readmission rates were detected between the groups [19]. The issue is the higher mean transvalvular pressure gradient after valve-in-valve implantation compared with the transvalvular pressure gradient after redo AVR. The higher transvalvular pressure gradient does not resolve the left ventricular hypertrophy, causing patient-prosthetic mismatch (PPM). PPM is well-known after AVR and is associated with poor survival rates and a higher incidence rate of congestive heart failure [22, 23]. A study by Duncan et al. showing that severe PPM after transcatheter valve-in-valve implantation did not affect 1-year mortality [24]. However, several surgical studies demonstrated that the adverse effects related to PPM occur at least 5 years after surgery. Thus, the conclusions of the Duncan study were premature. While PPM may be less relevant for octogenarian with failed bioprostheses, it is important in younger patients with > 10 year prognosis.

The outcomes for the current study were measured at 1-year after the intervention and may not be appropriate for young patients with longer life expectancies. We do not recommend that young patients receive valve-in-valve implantations to avoid the higher transvalvular pressure gradients. Patients who need either redo surgery or transcatheter intervention should be selected carefully, considering patients’ age, comorbidities, and anatomy suitable for transcatheter intervention [25].

A study comparing outcomes following transcatheter valve-in-valve implantations for failed stented and stentless aortic bioprostheses detected no significant differences in 30-day mortality (6.6% vs 4.4%; *P* = 0.12) or 1-year mortality (15.8% vs 12.6%; *P* = 0.15) between the groups. However, more periprocedural complications, including device migration, coronary obstruction and more than mild paravalvular leakage, were observed in valve-in-valve implantations for failed stentless aortic bioprostheses compared with complications after failed stented bioprostheses [24]. In addition, coronary obstruction occurred more frequently in patients with stentless or stented bioprostheses with externally mounted leaflets [26].

The PARTNER 2 registry showed 3-year outcomes following valve-in-valve implantations for degenerative aortic bioprostheses. The mean gradient at 3 years was 17 mmHg and 14.1% of patients had New York Heart Association class III or IV symptoms; New York Heart Association decreased baseline in 90.4% of patients. Despite the presence of a persistent mean gradient, left ventricular mass index decreased significantly at 3 years. The 3-year overall and cardiac mortality rates were 32.7% and 20.5%, respectively [27]. Although the data are encouraging for the elderly or high-risk patients, the data does not support valve-in-valve implantation instead of redo surgery for younger patients.

A study consisting of 148 patients with a mean age of 68 years revealed 5- and 10- year survival rates of 74.1% and 58%, respectively, after redo AVR for various reasons. The authors concluded that redo AVR results in acceptable late outcomes and only a minority of patients progress to valve-in-valve percutaneous implantations as a third intervention [28]. These results are similar to the present study.

## Limitations

There are several limitations in this study. First, this study is retrospective in nature. The pre/post-operative managements, surgical techniques, and patient selection bias may have affected the outcomes. Second, our study included a relatively small number of patients who underwent redo aortic interventions. The stentless group consisted of only 12 patients who underwent either redo surgeries or transcatheter valve-in-valve implantations. In addition, the stentless group received the greater proportion of transcatheter valve-in-valve implantations as opposed to the stented group. This might be responsible for the greater incidence of thromboembolic events in stentless group. Third, the redo surgery differed so greatly in terms of type of implanted valves and the concomitant procedures, leading to difficulty in direct comparison and assessing the durability. Thus, the study may not have been adequately powered to draw conclusions. However, we demonstrated similar early and mid-term outcomes following either redo surgery or transcatheter intervention for patients with failing stented or stentless bioprostheses. The concept of comparing outcomes between groups in the current era of transcatheter intervention is interesting. Our findings may be helpful in deciding which patients require redo surgery versus transcatheter intervention in the context of stented or stentless aortic bioprostheses.

## Conclusions

Aside from thromboembolism, no significant differences in early and mid-term outcomes after aortic valve interventions were detected in patients with stented or stentless valves. Redo surgery for the stentless group was challenging because most patients required root replacement. A transcathter valve-in-valve procedure may be a viable option for this cohort in the absence of infection. Redo AVR is likely a good option for infective endocarditis.

## Data Availability

The datasets used and/or analyzed during the current study are available from the corresponding author on reasonable request.

## Abbreviations

AVR: Aortic valve replacement
CABG: Coronary artery bypass grafting
NYHA: New York Heart Association
PVE: Prosthetic valve endocarditis
SVD: Structural valve deterioration
